# Observations on Fever

**Published:** 1828-10

**Authors:** W. F. Bow


FEVER.

### *Observations on Fever.* By W. F. Bow, M.D.

I HAD the honour of appearing in the Numbers of your Journal for March, April, and May, as the author of some speculations on the nature of fever. I there hazarded an opinion regarding the heat of the surface. I said that, during the first stage, the products of secretion throughout the system were imperfectly secreted, in consequence of the impairment of nervous energy; that these imperfect products at length irritated the extremities of the sentient nerves, and thus caused the reaction which introduced the second stage; that, owing to the great sensibility of the skin, reaction first manifested itself there by a sense of heat; and that the preternatural heat of fever depended on such irritation, and was not caused by increased vascular action.

My object in now addressing you is to call the attention of your numerous readers to this point: many of them may have an opportunity of watching the progress of febrile attacks in their own persons during the autumn: if so, I am confident they will find that heat of surface, more or less, invariably precedes increase either of force or frequency of pulse. I begin to suspect that the contrary opinion is the offspring of theory rather than of clinical observation, and that the general belief in this doctrine is the chief obstacle in our research into the nature of fever. As an instance how apt we are to be misled by preconceived ideas, or by the authority of great names, Dr. CURRIE, throughout his "Reports," never doubts but that the heat of the surface in fever is caused by increased vascular action. He believes it to accumulate first about the heart, and to extend to the surface; yet he commences his work with a case which shews evidence to the contrary. This case is the more to be depended on as it is reported by the patient himself, Dr. WRIGHT.

"By my attention to the sick man, I caught the contagion, and began to be indisposed on the 5th of September; and the following is a narrative of my case, extracted from notes daily marked down, &c. September 5, 6, 7.—Small rigors now and then; a *preternatural heat of the skin;* a dull pain in the forehead; the pulse *small and quick.*"

Dr. Good considers heat to be the result of increased action. "For as the former [heat] is the result of increased action, till such increased action takes place, the heat, as in the first stage of the paroxysm, may continue even below the natural standard."* Yet his diagnosis of mild remittent fever runs as follows: "The patient complains of drowsiness, and feels languid; is occasionally chilly, and afterwards flushed, but without perspiration; for the skin is *hot* and *dry,* the thirst considerable, commonly with nausea and a total loss of appetite. In the course of the day, but usually towards the evening, the *pulse quickens,* the *heat increases,* and at length terminates in sweat."

Dr. Good also illustrates the autumnal remittent with a case, in which I suspect heat of skin existed, with a *feeble* pulse.

"In the case of a young lady, in her seventeenth year, whom I lately attended, the attack was slight; and no serious evil was at first apprehended. The pulse was about ninety in a minute, and rather *small;* the bowels were relaxed, the motions bilious, and the stomach suffered from nausea. A gentle emetic seemed to afford some relief to the stomach, and a dose of rhubarb and calomel to the bowels; but the *fever* continued with a daily and increasing exacerbation, for the most part at mid-day, or soon after. The stomach again became irritable and sick, and the sickness was again connected with diarrhœa; but the stools were colourless and watery, and nothing was rejected from the stomach but the diluent food that was swallowed. The skin was now *very* hot and dry; the pulse from a hundred to a hundred and twenty strokes in a minute."

Now, in the first part of this case, although the heat of the skin is not mentioned, there must have been considerable heat, because Dr. Good says "the fever continued." Without heat greater than natural, the disease could not have been recognised as fever; and a pulse ninety, and smaller than natural, certainly cannot indicate increased action of the sanguiferous system.

*Alnwick; August* 20, 1828.

* Study of Medicine, vol. ii. p. 40.

